# Reaction-Based, Fluorescent Film Deposition from Dopamine and a Diamine-Tethered, Bis–Resorcinol Coupler

**DOI:** 10.3390/ijms20184532

**Published:** 2019-09-13

**Authors:** Maria Laura Alfieri, Mariagrazia Iacomino, Alessandra Napolitano, Marco d’Ischia

**Affiliations:** Department of Chemical Sciences, University of Naples Federico II, Via Cintia 4, 80126, Naples, Italy; marialaura.alfieri@unina.it (M.L.A.); iacomino.mg@gmail.com (M.I.); dischia@unina.it (M.d.)

**Keywords:** dopamine, hexamethylenediamine, hydrophobic to hydrophilic switch bis-resorcinol coupler, fluorescent coating, strong fluorescence emission, yellow chromophore

## Abstract

The reaction-based deposition on various surfaces of an all-organic fluorescent coating is reported here, involving autoxidation of 2 mM dopamine in carbonate buffer at pH 9.0, in the presence of a 1 mM diamine–resorcinol coupler (Bis–Res) prepared from 2,4-dihydroxybenzaldehyde and hexamethylenediamine (HMDA). Spectral analysis of the films coupled with an LC-MS investigation of the yellow fluorescent mixture was compatible with the formation and deposition of HMDA-linked methanobenzofuroazocinone fluorophores. Both the emission properties and hydrophobicity of the film were abated in a reversible manner following exposure to acid vapors. These results provide an entry to efficient and practical fluorescent coating methodologies based on in situ generation and the deposition of wet adhesive, as well as fluorescent materials combining a strongly emitting fluorophore with the film-forming properties of long chain diamines.

## 1. Introduction

Fluorescent thin films and coatings may provide useful tools for a variety of biomedical, environmental, and technological applications with special reference to specific sensing materials operating both in vapor phase (e.g., volatile amines, illicit drugs, nitrotoluenes) [1,2] and in solution (e.g., Hg^+^, F^−^, Au^3+^) [3]. Additional perspectives for the use of fluorescent films include real-time monitoring of cell growth and metabolic changes, specific input-responsive packaging, and damage detection in cultural heritage, to mention only some. The repertoire of approaches and technologies for fluorescent thin films and coatings is still expanding, and is comprised of aggregation-induced emission materials [4,5,6,7], derivatization of photochemically stable fluorophores into low-molecular mass gelators [8,9], and high quantum yield carbon dots [10,11,12]. In representative examples from the past few years, fluorescence is carried by a stable fluorophore, such as modified naphthalene diimide [9]; perylene bisimide (PBI) [13]; a Schiff base derivative [14]; rhodamine derivatives grafted onto polymers [15]; chlorophyll [16]; and quantum dots or by organic–inorganic complexes based, e.g., on Eu^3+^/polyhydroxybutyrate [17]. Relatively few cases deal with all-organic films [16], and little precedent is available, to the best of our knowledge, about the in situ deposition of all-organic, reaction-based fluorescent films and coatings by wet dipping technologies. 

**Scheme 1:**
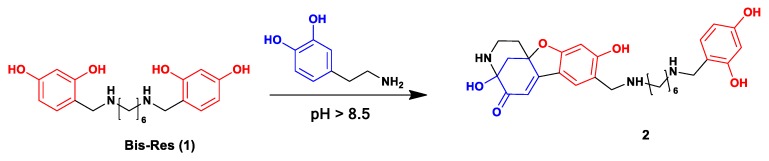
Rationale underlying the development of hexamethylenediamine (HMDA)–resorcinol conjugate (Bis-Res) dopamine coupling for fluorescent coating technology.

In this paper, we disclose a strategy for the in situ development of a highly adhesive and strongly emitting, all-organic film-forming system via the autoxidation of dopamine in the presence of a cross-linking, hexamethylenediamine (HMDA)–resorcinol conjugate referred to as Bis–Res (Scheme 1).

The chemistry outlined in Scheme 1 relies on the well-known dopamine quinone–resorcinol coupling reaction, leading to a highly fluorescent methanobenzofuroazocinone system of azamonardine and related natural fluorophores [18,19,20,21,22]. Recent investigation of this chemistry led us to develop a unimolecular variant of this reaction, referred to as “FluoResCat”, which may provide a promising tool for pH-sensitive oxygen and volatile amine sensing [23]. As an extension of this reaction, we have now probed the deposition of fluorescent films, capitalizing on the crosslinking properties of long chain diamines like hexamethylenediamine (HMDA) [24,25,26]. 

HMDA, regarded as a lysine mimic in mussel-inspired adhesive systems [27], has been shown to enable the deposition of adhesive films from gallic acid, caffeic acid, 5,6-dihydroxyindole [26], *N*-protected DOPA [28], and other catechol-containing polymers [29,30,31]. This versatile strategy provides an entry to the design of a variety of functional films and coatings, including, e.g., antioxidant properties via binding to melanin-related metabolites [32]. Different from the coupling of HMDA to catechol and catecholamines, which may occur *via* Michael addition or condensation reactions at the *o*-quinone intermediates [25,28], the chemistry leading to the fluorescent methanobenzofuroazocinone system from dopamine and resorcinol relies on the high nucleophilic reactivity of the carbon position of resorcinol, which efficiently competes with the primary amino group of the catecholamine, inhibiting the cyclization pathway. 

## 2. Results

A symmetrical bis-resorcinol derivative (Bis–Res), in which the two aromatic rings are held together by an HMDA tether, was synthesized from 2,4-dihydroxybenzaldehyde *via* Schiff base formation and reduction (Scheme S1). 

**Figure 1:**
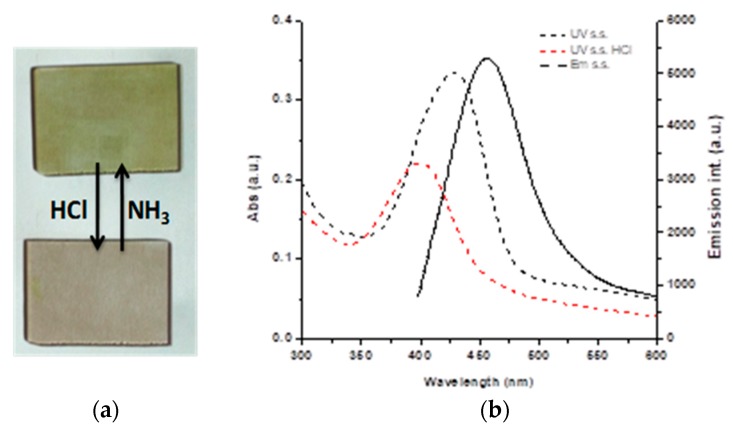
Picture (**a**) and UV-VIS and fluorescence spectra (**b**) of a dopamine/Bis–Res coated quartz at 6 h reaction time, before and after exposure to HCl vapors.

Stirring 2 mM dopamine with 1 mM Bis–Res in carbonate buffer at pH 9.0 led to the gradual development of an intensely emitting yellow chromophore, accompanied by the deposition of a fluorescent thin film on a quartz substrate immersed in the reaction flask (Figure 1).

Profilometry data (Table 1) indicates a film thickness of 55 ± 2.7 nm, with a roughness of 15 nm. 

**Figure 2:**
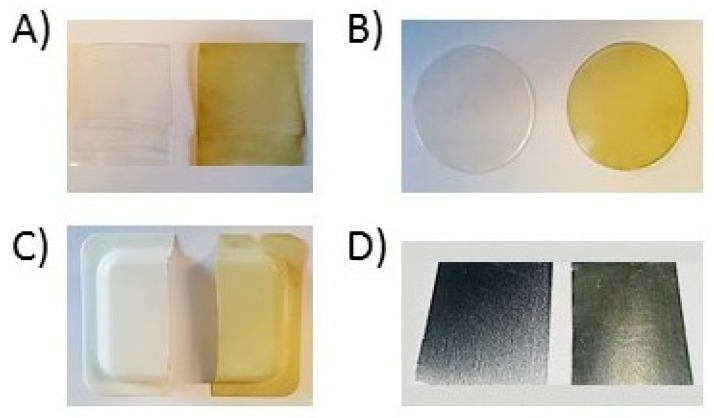
Bis–Res/DA coatings on various materials: (**A**) polyethylene, (**B**) polycarbonate, (**C**) polystyrene, and (**D**) aluminium. On the left of each are uncoated materials.

Efficient film formation was also observed on a variety of materials immersed in the oxidation mixture of dopamine and Bis–Res (Figure 2).

**Figure 3:**
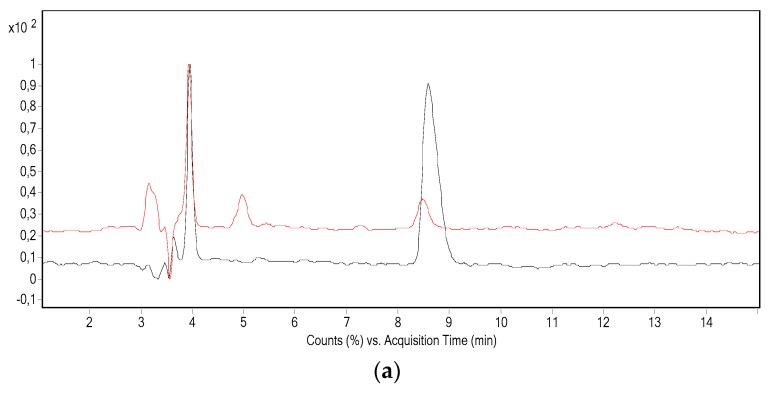

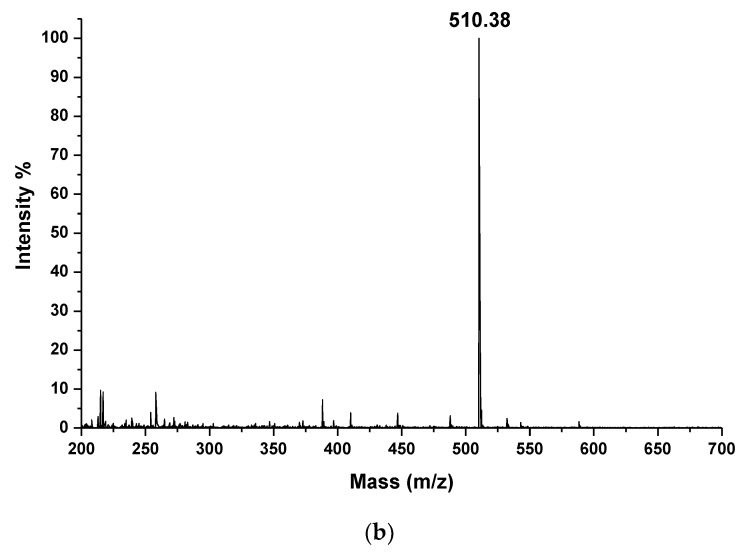
LC-MS analysis of the reaction mixture of Bis–Res (*R_t_* = 8.9 min) and dopamine (*R_t_* = 4 min). (**a**) total ion current chromatogram after 0 min (black) and 150 min (red). (**b**) mass spectrum of the peak eluting at 5.1 min, base peak [M + H]^+^ = m/z 510.4, eluting system = 0.1% HCOOH—MeOH 8:2.

Spectrophotometric analysis of the coating in the solid state revealed an absorption maximum at 420 nm and an intense emission at 460 nm, whose features matched those of the dopamine–resorcinol adduct. In support of this conclusion, LC-MS analysis of the reaction mixture (Figure 3) revealed the formation of a main species with m/z = 510 [M+H]^+^, consistent with the expected coupling for product **2** depicted in Scheme 1. A diadduct involving both resorcinol moieties of **1** could be considered as an additional reaction product. However, under the conditions adopted, Bis–Res **1** and dopamine are not completely consumed (Figure 3, red line), and hence the diadduct would be expected to represent a minor product.

**Figure 4:**
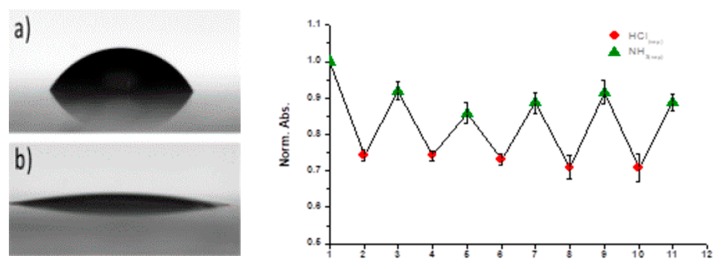
Left: water droplet on the Bis–Res/dopamine coating before (**a**) and after (**b**) exposure to HCl vapors. Right: relative absorbance changes at 420 nm of Bis–Res/dopamine film following repeated sequential exposure to HCl and NH_3_ vapors.

Interestingly, exposure of the coated substrate to HCl vapors caused an apparent discoloration of the film with a consistent hypsochromic shift, a marked quenching of fluorescence, and a drop of the water contact angle (WCA) of the film from about 50° to values below instrumental limits, indicating conversion into a superhydrophilic material (Figure 4, left). The HCl-induced absorption shift proved to be completely reversible upon exposure to gaseous ammonia over at least five cycles (Figure 4, right). The marked drop in the WCA value was attributed to the protonation of the amine residues linked to the methanobenzofuroazocinone scaffold in the adduct, with the generation of positively-charged sites increasing the hydrophilic character of the film.

**Figure 5:**
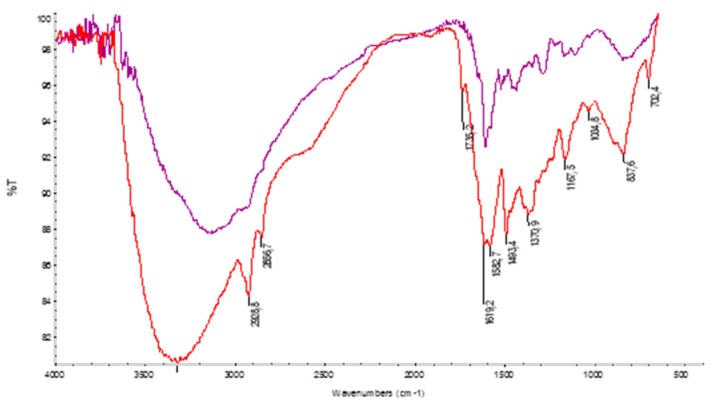
ATR/FT-IR of the dopamine/Bis–Res coated aluminum slide before (red line) and after (violet line) exposure to HCl vapors.

The origin of superhydrophilicity following exposure to HCl vapors was then investigated by ATR/FT-IR analysis. Comparison of the spectra of the film deposited on aluminum substrate before and after acid treatment indicated (1) a detectable shift of the broad N–H and O–H stretching band from ca. 3300 cm^−1^ to ca. 3100 cm^−1^; and (2) marked changes in the low energy bands, with the development of two weak bands at 1560 cm^−1^ and 1200 cm^−1^ (Figure 5). Though it is difficult to assign these latter bands with certainty, it is worth mentioning the well-known appearance in the spectra of protonated secondary amines of bands around 1590 cm^−1^, which are missing in the free base [33]. Support to the proposed assignment could derive from the application of *spectral-inverse quantum* methodology, recently developed for the interpretation of the IR spectral features of solid materials like mesoporous silica gel [34]. 

## 3. Discussion

Emitting film deposition by fluorescence turn on processes is a promising approach to surface functionalization that is not adequately represented in the literature, to the best of our knowledge. The present proof-of-concept investigation into the autoxidation-promoted reaction of dopamine with the Bis–Res coupler demonstrates the potential of this chemistry to produce novel adhesive species that can coat various substrates with acid-tunable emission and surface properties. Distinctive outcomes of this study, which deserve further investigation, include:

1) The demonstration of the uncommon emission properties of the dopamine–resorcinol coupling product in the solid state as thin film;
2) Verification of the role of HMDA as an almost universal coupler, inducing film deposition under a variety of conditions;
3) The added value of including, in the film-forming adducts, strongly basic secondary amine sites susceptible of reversible protonation, to serve as on–off switches for surface property modification.

Ongoing work is directed (1) to fine tuning the surface and emission properties of the films via modification of the Bis-Res coupler, in order to introduce additional functional groups on both the diamine and resorcinol moieties; (2) to gaining better insight into the structural properties of the film in relation to the current knowledge of underwater adhesion mechanisms; and (3) to assessing the potential of the fluorescent films for biomedical and nanotechnological applications, e.g., for stem cell growth and theranostics. 

## 4. Materials and Methods 

All reagents were obtained from commercial sources and used without further purification. Organic solvents were used as purchased. Water was of MilliQ^®^ quality. Buffers were prepared by standard procedures. A Crison pH-meter equipped with a 5014 Crison electrode was used for pH measurements at room temperature. Analytical thin-layer chromatography (TLC) was performed on 0.25 mm thick pre-coated silica gel plates (60 F254). UV-VIS absorption spectra were registered at room temperature on a V-560 JASCO spectrophotometer, using calibrated 2 mL quartz cuvettes, and for quartz slides, on a Cary 4000 UV-Vis spectrophotometer, using the solid sample holder. Steady-state fluorescence emission spectra were recorded with a FP-750 JASCO spectrofluorometer. ^1^H-NMR and ^13^C-NMR spectra were recorded in deuterated solvents at 400 MHz on a Bruker DRX 400; δ values are reported in ppm and coupling constants are given in Hz. HPLC analyses for reaction monitoring were performed on an Agilent 1100 series instrument equipped with an LC-10AD VP pump and a G1314A UV-VIS detector, using a Sphereclone C18 column (4.6 × 150 mm, 5 μm). LC-MS analysis was conducted on an ESI-TOF 1260/6230DA (Agilent Technologies) in positive ion mode. IR spectra were recorded on Nicolet 5700 FT-IR + Smart performer spectrometer, mounting a Continuum FT-IR Microscope and on Bruker Optics TENSOR 27 FT-IR.

### 4.1. Synthesis and Structural Characterization of Bis–Res 

A mixture of hexamethylenediamine (1.81 mmol, 400 mg) and 2,4-dihydroxybenzaldehyde (3.62 mmol, 950 mg) in absolute ethanol (35 mL) was stirred at room temperature for 1 h. The yellow solid that separated was centrifuged three times at 5000 rpm and washed with cold ethanol, then dried in vacuo to give the Schiff base (**1a**, 90% yield). The latter was then rinsed in glacial acetic acid (10 mM, 160 mL) and reduced by NaBH_4_ (16 mmol, 605 mg). When hydrogen evolution ceased, 3 M HCl was added until the pH was 5.0 The mixture was dried under vacuum, then rinsed in 1 mL of water before desalting on Sephadex G-10 resin, using HCl 0.05 M as the eluant. Ten fractions of 8 mL each were collected and analyzed by UV-VIS spectrometry. After removal of the acid under reduced pressure, compound 1 was obtained as di-hydrochloride salt in the form of a brownish orange solid (yield = 75%).

(**1a**) Rf = 0.27 (CHCl_3_-MeOH 9:1); UV-VIS: λ_max_ (MeOH) = 301 nm (ε= 33410 L•mol^−1^•cm^−1^) and 371 nm (ε = 15879 L•mol^−1^•cm^−1^); m/z = 357.18 [M+H]^+^; ^1^H NMR (400 MHz, DMSO-d_6_) δ 8.30 (s), 7.13 (*d*, J = 8.5 Hz), 6.22 (*d*, J = 8.4 Hz), 6.13 (s), 3.48 (*t*, J = 6.6 Hz), 1.59 (br. m), and 1.36 (br. m) (Figure S1).^13^C NMR (101 MHz, DMSO–d_6_) δ 165.7, 164.5, 162.3, 133.0, 111.1, 106.7, 102.9, 56.4, 30.4, and 26.1 (Figure S2). The resonance assignment was deduced from two-dimensional (2D) NMR analysis. ATR (cm^−1^) = 1227 (phenolic C–O stretch), 1358 (phenolic C–O bend), 1637 (C=N stretch), 2932 (aliphatic C-H bend and stretch), 3065 (aromatic C–H stretches), and 3509–3638 (phenolic O–H stretches).

(1b) UV-VIS: λ_max_ (MeOH) = 279 nm (ε= 4944 L•mol^−1^•cm^−1^); m/z: 361.21 [M+H]^+^. ^1^H NMR (400 MHz, MeOH-d_4_) δ 7.12 (*d*, J = 8.2 Hz), 6.39 (s), 6.33 (*d*, J = 6.8 Hz), 4.09 (s), 3.01–2.93 (br. m), 1.73 (br. m), and 1.43 (br. m) (Figure S3); ^13^C NMR (101 MHz, MeOD-d_4_) δ 161.4, 158.6, 133.5, 109.8, 108.1, 103.4, 47.8, 47.6, 27.1, and 26.6 (Figure S7). The resonance assignment was deduced from 2D NMR analysis (Figures S4–S6). ATR-FT/IR (cm^−1^) = 1172 (C–N stretch), 1593 (N–H bend), 2796–2935 (aliphatic C–H bend and stretch), 3041 (aromatic C–H stretches), 3235–3292 (N–H stretch), and 3531–3622 (phenolic O–H stretches) (Scheme S8).

### 4.2. General Procedure for the Oxidative Coupling of Compound **1** with Dopamine

The reaction was carried out using compound **1** dihydrochloride at 1 mM and dopamine at 2 mM in 0.05 M sodium carbonate buffer, pH 9.0, under stirring in air. Typically, the reaction was carried out on a 50 mL volume. After complete dissolution of the reagents in the buffer, a neat substrate (glass or quartz coverslips rinsed with piranha mixture H_2_SO_4_:H_2_O_2_ 5:1, polyethylene or polystyrene cuts, polycarbonate slides, or aluminum foils) was immersed in the reaction mixture for 6 h. The development of a blue fluorescence was rapidly observed, followed by deposition of a thin layer of organic material on the substrate and concomitant separation of a brownish-green solid. The course of the reaction was followed by LC-MS analysis. After 2 h reaction time, the liquid chromatography (LC) profile of the reaction mixture showed substantial consumption of dopamine and compound **1**, as well as the formation of a main component with a pseudomolecular ion peak corresponding to the monoadduct **2**, shown in Scheme 1. The quartz substrates after coating were subjected to repeated ultrasound-assisted washing in water before spectrophotometric/fluorimetric analysis. All other substrates were repeatedly washed with deionized water and dried in air. 

## 5. Conclusions

The autoxidation of dopamine in carbonate buffer at pH 9.0 in the presence of the cross-linking coupler Bis–Res is shown herein to give rise to a film-forming coupling product with a detectable fluorescence in the solid state. Exposure of the film to acid vapors causes a marked loss of fluorescence and a drop in WCA, suggesting amine protonation-induced superhydrophilicity.

These results provide a new rationale toward the development of the first class of bioinspired, all-organic, reaction-based turn-on fluorescent films for smart coating applications. The reported chemistry is amenable to tailored modifications and the tuning of properties via the proper selection of catecholamine and Bis–Res derivatives.

## Figures and Tables

**Table 1:** Profilometry data of the Bis–Res/dopamine (DA) coating on glass before and after HCl_(vap)_ exposure.

Coating	λ_max/ex_ (nm)	λ_em_ (nm)	Thickness (nm)	Roughness (nm)	Water Contact Angle (deg)
**Bis-Res/DA**	420	464	55 ± 2.7	15	52 ± 3.6
**Bis-Res/DA-HCl**	400	no	48 ± 0.2	16	Not calculable

## Supplementary Materials

Supplementary materials can be found at https://www.mdpi.com/1422-0067/20/18/4532/s1.

## Abbreviations

HMDA	Hexamethylenediamine
Res	Resorcinol
WCA	Water contact angle
