# Supplementary material for: Reaction-Based, Fluorescent Film Deposition from Dopamine and a Diamine-Tethered, Bis–Resorcinol Coupler
Source: Int J Mol Sci. 2019 Sep 13;20(18):4532. doi: 10.3390/ijms20184532 (PMC6769982; doi:10.3390/ijms20184532)
Supplement: Supplementary file 1 [file ijms-20-04532-s001.pdf]

Supporting Information

Reaction-Based, Fluorescent Film Deposition from Dopamine and a Diamine-Tethered, Bis–Resorcinol Coupler

.

Maria Laura Alfieri[1], Mariagrazia Iacomino[1], Alessandra Napolitano[1,*] and Marco d'Ischia[1]

[1] Department of Chemical Sciences, University of Naples Federico II, Via Cintia 4, 80126, Naples, Italy; marialaura.alfieri@unina.it; iacomino.mg@gmail.com; alesnapo@unina.it; dischia@unina.it.

* Correspondence: alesnapo@unina.it.

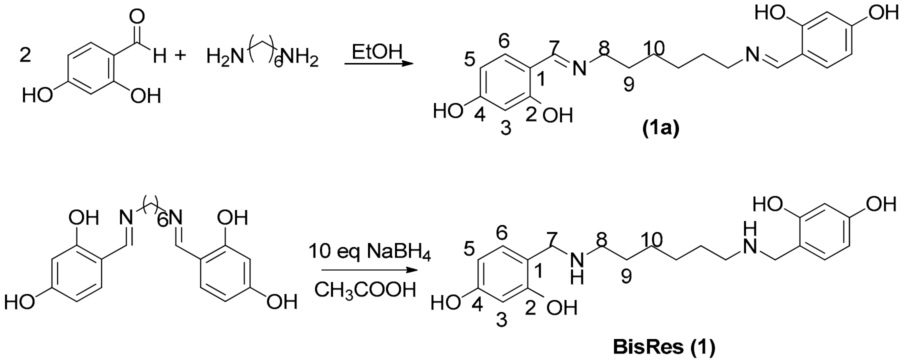

Scheme S1. Synthetic route to BisRes (**1**).

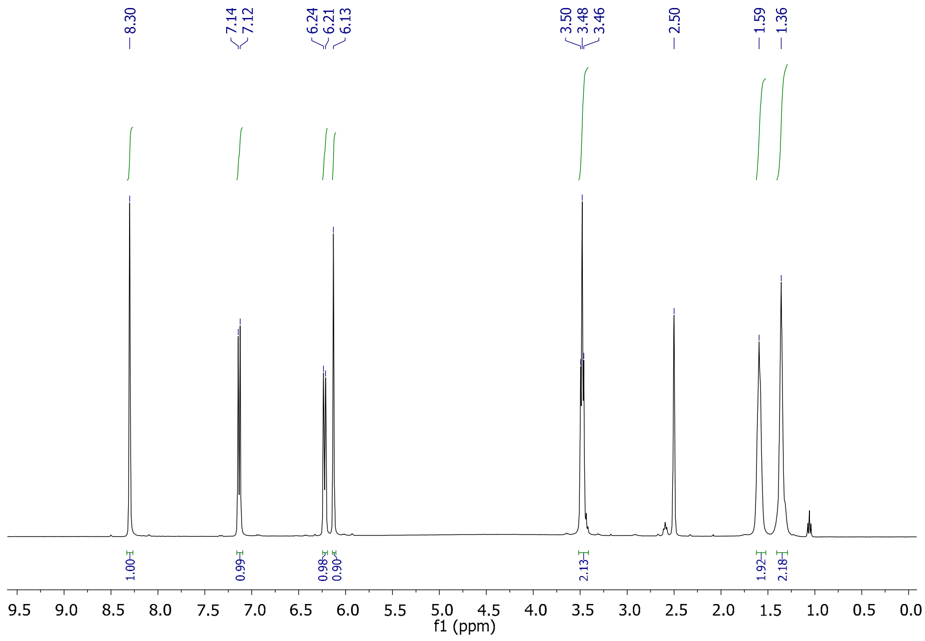

Figure S1.^{1}H NMR (400 MHz, DMSO-d_6) of the Schiff base (**1a**) δ 8.30 (s, H-7), 7.13 (d, *J* = 8.5 Hz, H-6), 6.22 (d, *J* = 8.4 Hz, H-5), 6.13 (s, H-3), 3.48 (t, *J* = 6.6 Hz, H-8), 1.59 (br. m, H-9), 1.36 (br. m, H-10).

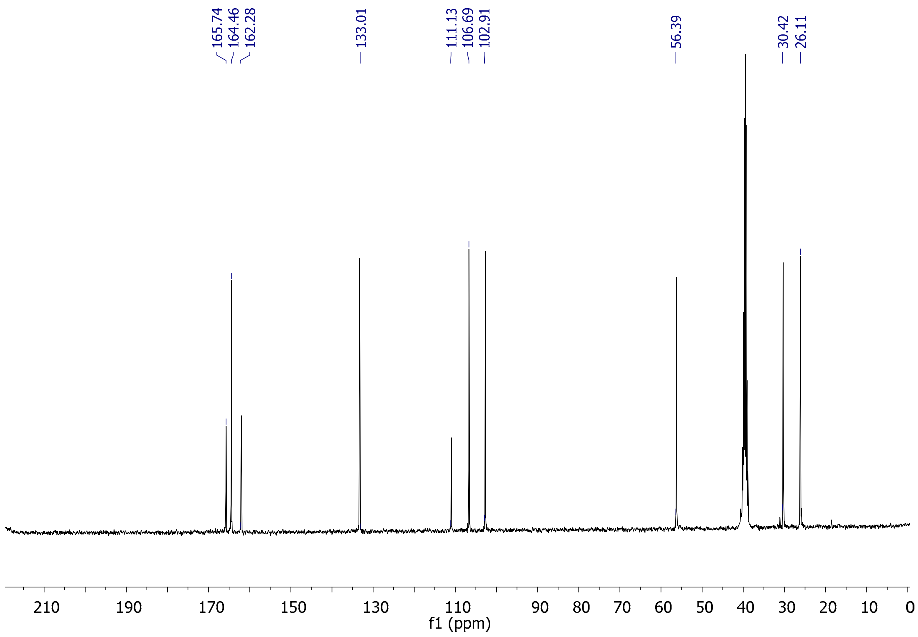

Figure S2. ^{13}C NMR (101 MHz, DMSO-d_6) of the Schiff base (**1a**).

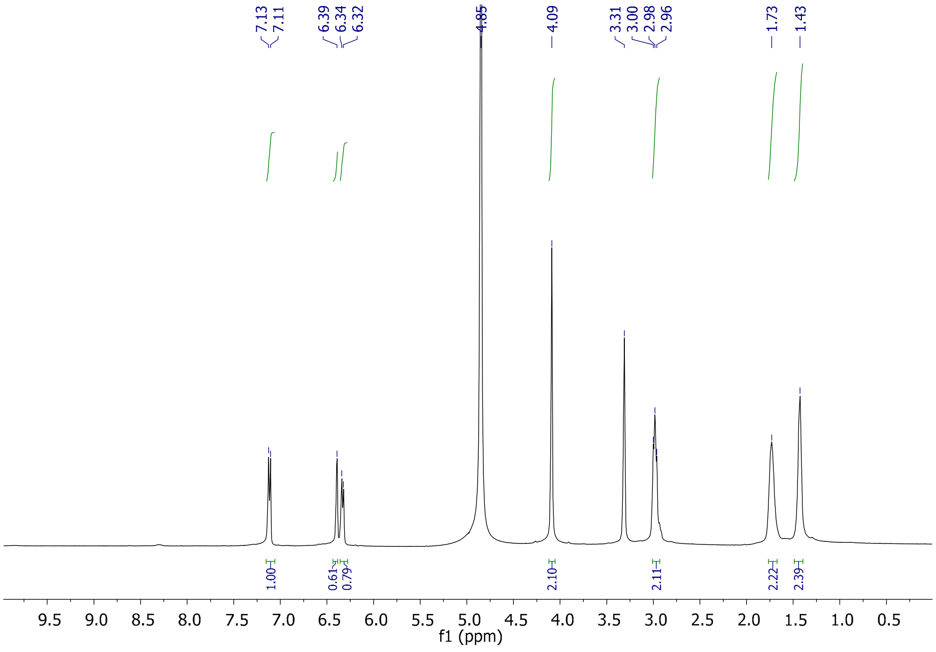

Figure S3. ^{1}H NMR (400 MHz, MeOH-d_4) of **1** δ 7.12 (d, *J* = 8.2 Hz, H-6), 6.39 (s, H-3), 6.33 (d, *J* = 6.8 Hz, H-5), 4.09 (s, H-7), 3.01 – 2.93 (br. m, H-8), 1.73 (br. m, H-9), 1.43 (br. m, H-10).Resonance assignment follows from analysis of ^{1}H,^{1}H COSY spectrum.

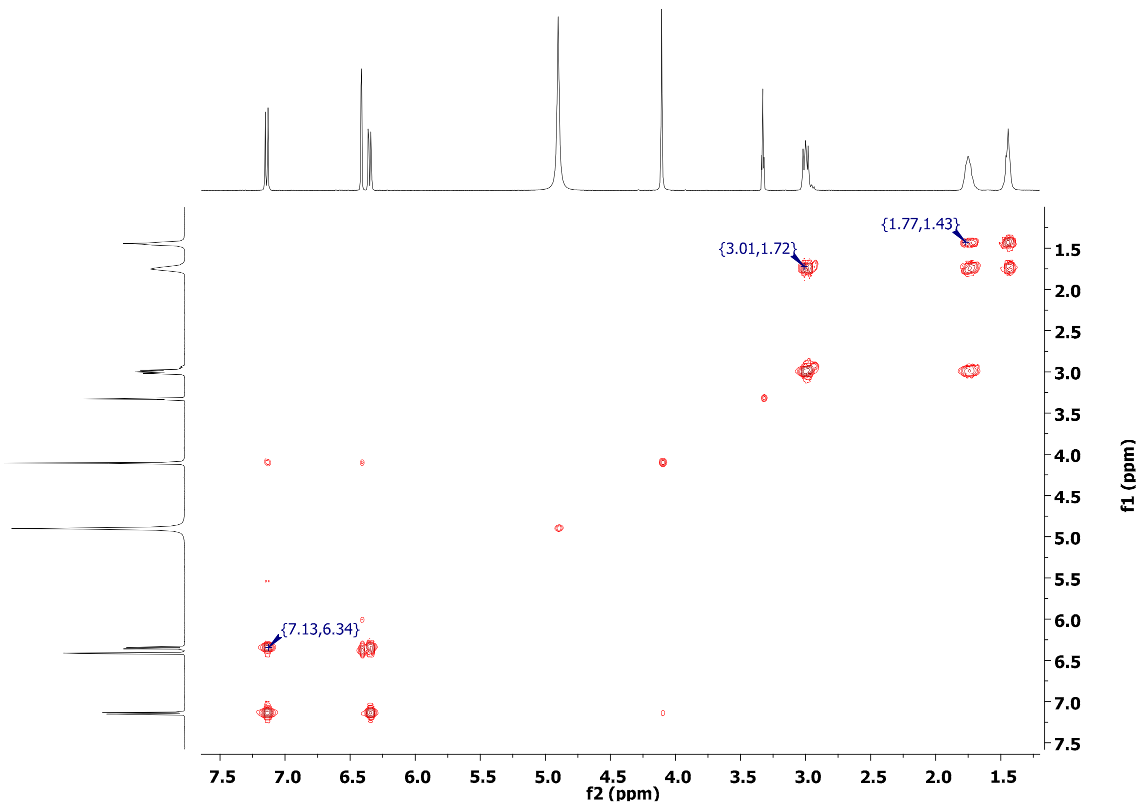

Figure S4. ^{1}H, ^{1}H COSY spectrum of **1** (400 MHz, MeOH-d_4)

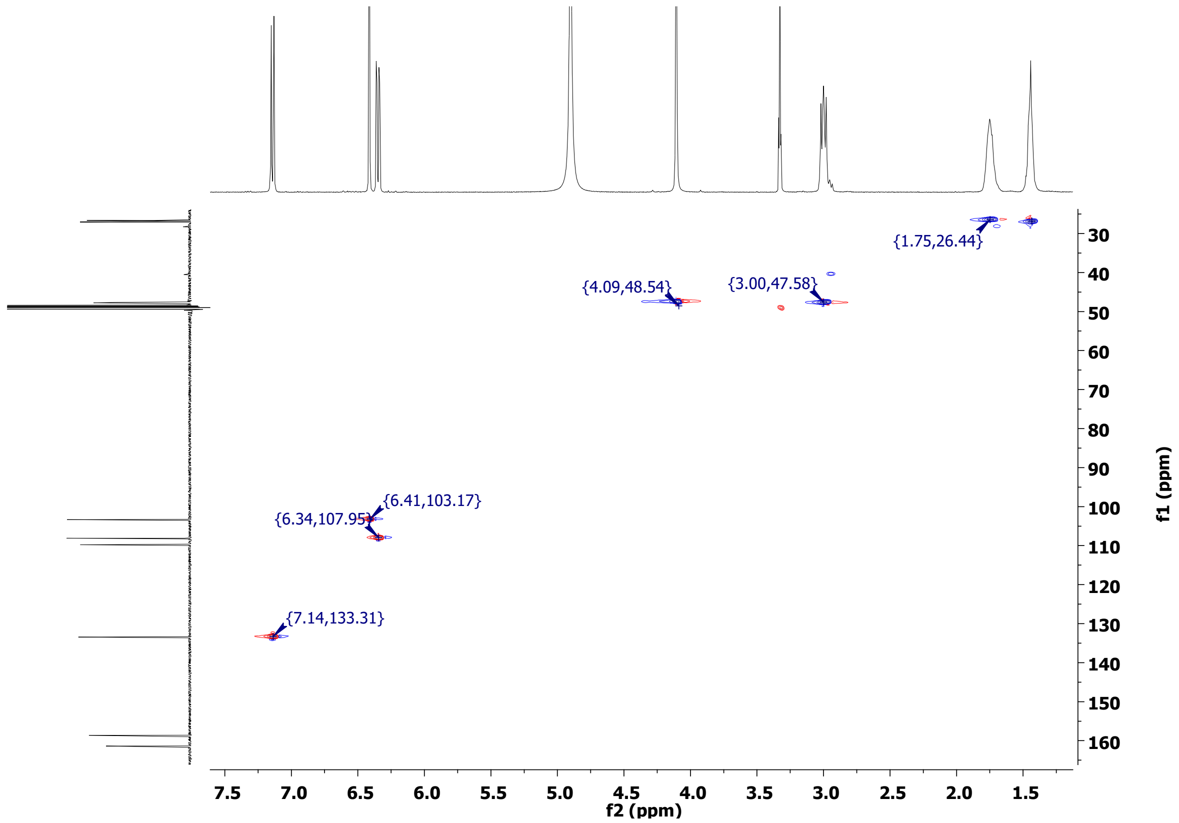

Figure S5. ^{1}H, ^{13}C HSQC spectrum of **1** (400 MHz, MeOH-d_4)

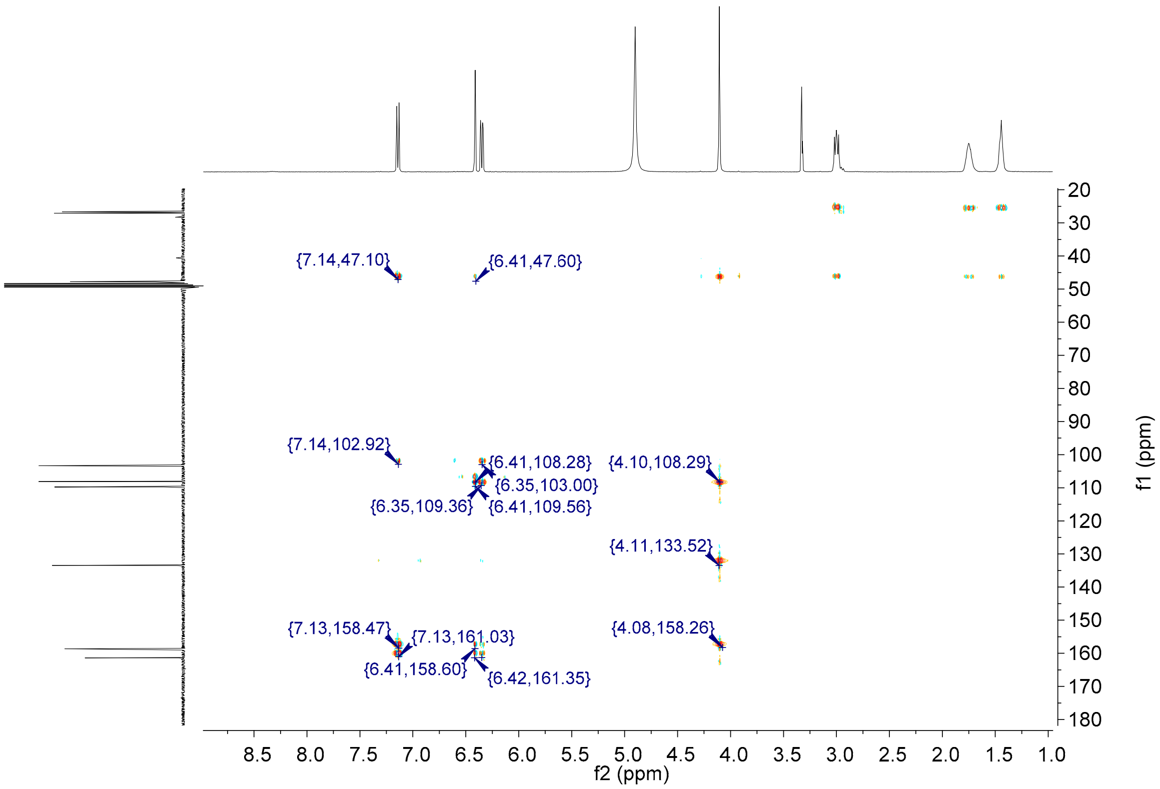

Figure S6. ^{1}H, ^{13}C HMBC spectrum of **1** (400 MHz, MeOH-d$_4$).

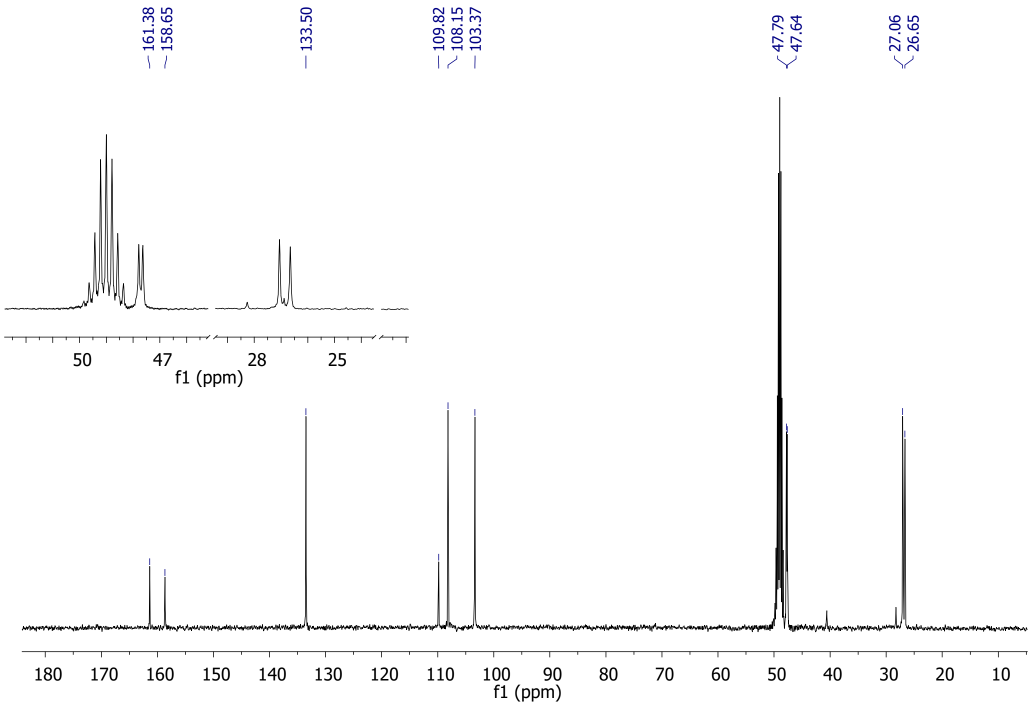

Figure S7.^{13}C NMR (101 MHz, MeOD-d$_4$) of **1** δ 161.4 (C-4), 158.6 (C-2), 133.5 (C-6), 109.8 (C-1), 108.1 (C-5), 103.4 (C-3), 47.8 (C-7), 47.6 (C-8), 27.1 (C-9), 26.6 (C-10).

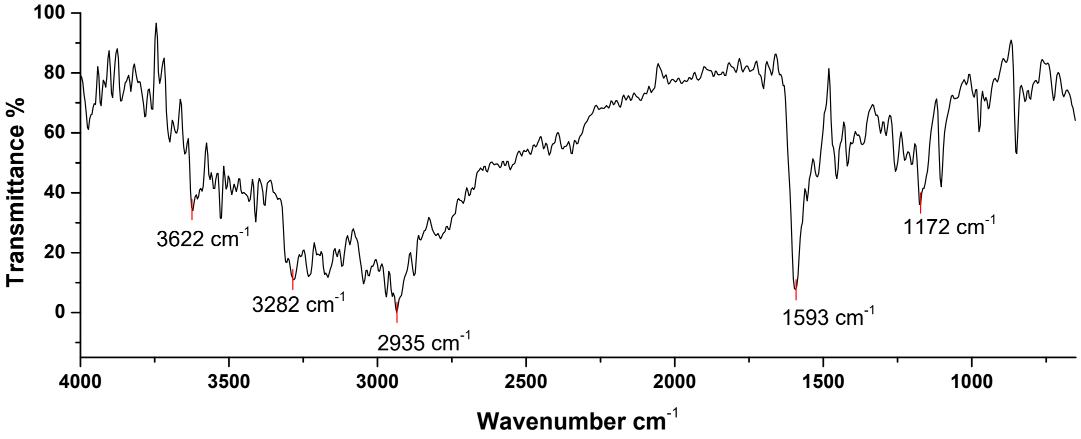

Figure S8. ATR/FT-IR of **1** (cm^{-1})1172 (C-N stretch), 1593 (N-H bend), 2796-2935 (aliphatic C-H bend and stretch), 3041 (aromatic C-H stretches), 3235-3292 (N-H stretch), 3531-3622 (phenolic O-H stretches).
